# Tyrosine kinase inhibitors for chronic myeloid leukemia: a landscape analysis of global clinical trials based on public registries

**DOI:** 10.3389/fphar.2026.1904028

**Published:** 2026-09-07

**Authors:** Lichuan Zhang, Qingyuan Tian, Yalin Tang, Hangyuan Liu, Yaxin Chen, Haiquan Wen, Jing Liu, Xinru Zhang, Rong Huang, Zining Luo, Tianxiang Geng, Jiebin Xie

**Affiliations:** 1 Department of Gastrointestinal Surgery, Affiliated Hospital of North Sichuan Medical College, Nanchong, Sichuan, China; 2 School of Clinical Medicine, North Sichuan Medical College, Nanchong, Sichuan, China; 3 School of Stomatology, North Sichuan Medical College, Nanchong, Sichuan, China; 4 Department of Orthopedics, Yantai Yuhuangding Hospital Affiliated to Medical College of Qingdao University, Yantai, Shandong, China

**Keywords:** chronic myeloid leukemia, clinical trials, drug, tretment, tyrosine kinase inhibitors

## Abstract

**Background:** Chronic myeloid leukemia is commonly treated with tyrosine kinase inhibitors, and numerous clinical trials have been conducted globally. However, the overall registration distribution, trial development patterns, and public availability of trial results remain unclear. This study aimed to map the global landscape of such trials and assess the public availability of their results.

**Methods:** This registry-based cross-sectional landscape analysis searched five major public clinical trial registries up to 19 March 2026. Results availability among eligible completed trials was assessed using registry records and a supplementary bibliographic search. Two reviewers independently screened trials, extracted data, and resolved discrepancies by adjudication. Descriptive statistical analysis was conducted using R software. We focused on completed trials finished for at least 2 years to evaluate publication.

**Results:** A total of 125 trials were included, of which 96 (76.8%) were interventional studies and 23 (18.4%) were randomized. Trials were concentrated in the United States, Europe, China, and Australia. Most trials targeted BCR-ABL1 and were Phase II studies, whereas Phase I and Phase IV studies were less common. Among 52 eligible completed pharmacological trials, publicly identifiable results were available for 25 trials (48.1%). Among the 44 trials with reported clinical phase, results availability was 77.8% (7/9) for Phase III trials and 0% (0/4) for Phase I trials. Results availability varied descriptively across drug groups, although no drug-specific comparison remained statistically significant after adjustment for multiple testing.

**Conclusion:** Global trials are geographically and developmentally unbalanced, with incomplete public availability of trial results. Future research should strengthen international collaboration, optimize trial phase distribution, support novel agent development, and promote timely and complete public disclosure of trial results.

## 1 Introduction

Chronic myeloid leukemia (CML) is a myeloproliferative neoplasm defined by the Philadelphia chromosome abnormality, resulting from the reciprocal translocation t (9; 22) (q34; q11.2) that generates the BCR-ABL1 fusion oncogene (Jabbour and Kantarjian, 2025). The global annual incidence of CML is approximately 2 per 100,000 population, with a relatively stable incidence trend. In the United States, approximately 9,300 individuals are newly diagnosed with CML annually, and about 150,000 individuals are estimated to be living with CML. Globally, approximately 5 million individuals are estimated to be living with CML (Jabbour and Kantarjian, 2025). With the widespread application of targeted therapy, the survival time of patients has been prolonged, leading to a slow increasing trend in the prevalent patient population (Jabbour and Kantarjian, 2025). The introduction of BCR-ABL1 tyrosine kinase inhibitors (TKIs) has reduced the annual mortality of CML from approximately 10%–20% in the pre-TKI era to about 1% (Kantarjian et al., 2025). At present, the research hotspots and frontiers in chronic myeloid leukemia (CML) focus primarily on three aspects: elucidation and reversal of TKI resistance mechanisms, prognostic improvement in patients with blast-phase CML (BP-CML), and enhancement of treatment-free remission (TFR) rates (Osman et al., 2021).

At present, ATP-competitive TKIs are recommended as the first-line standard therapy by both domestic and international guidelines for all newly diagnosed patients with chronic-phase chronic myeloid leukemia (CML-CP) (Cortes et al., 2026). Four tyrosine kinase inhibitors (TKIs), including imatinib, dasatinib, bosutinib and nilotinib, have been approved by the US Food and Drug Administration (FDA) for the first-line treatment of newly diagnosed chronic-phase chronic myeloid leukemia (CML-CP) (Jabbour and Kantarjian, 2024). The clinical application of TKIs has fundamentally revolutionized the treatment landscape of CML, transforming this fatal disorder into a manageable chronic disease, and the life expectancy of affected patients has approximated that of age-matched general populations. Long-term follow-up studies spanning 5–14 years, together with numerous short-term clinical investigations in newly diagnosed CML-CP patients, have further confirmed the durable and consistent long-term disease control efficacy of TKIs in CML (Cortes et al., 2026; Jabbour and Kantarjian, 2024; Guilhot and Hehlmann, 2025). Nevertheless, there are still unmet clinical needs in the first-line treatment of CML: more than 50% of newly diagnosed patients fail to achieve a major molecular response (MMR; defined as BCR::ABL1 transcript level on the International Scale (BCR::ABL1IS) ≤ 0.1% at 12 months) in clinical practice; the incidence of low-grade adverse events (AEs) that impair patients’ quality of life (QOL) and treatment adherence is relatively high during long-term TKI treatment. Given the above *status quo*, new first-line therapeutic options that can optimize disease control and quality of life are still warranted (Cortes et al., 2026). In recent years, novel agents represented by asciminib have been introduced into clinical research and application, which is the first STAMP inhibitor. Published early clinical data have shown that asciminib can be applied to the subsequent treatment of patients with TKI resistance or intolerance, so as to meet the relevant unmet clinical needs (Cortes and Lang, 2021). In addition, TKI-related hepatotoxicity is a key safety concern requiring close attention in clinical treatment. Available data have shown that nearly 50% of oral drugs available on the market carry hepatotoxicity-related risks (Wang et al., 2021). Collectively, although TKIs have effectively prolonged the survival of CML patients, a variety of treatment-related adverse reactions are inevitably accompanied during the treatment course (Atallah et al., 2021).

To date, numerous clinical trials have evaluated the efficacy and safety of TKIs for the treatment of CML. Relevant data have been stored in public clinical trial databases and registration systems, such as ClinicalTrials.gov, the EU Clinical Trials Register, and the Chinese Clinical Trial Registry. Nevertheless, several studies are still designed as single-center and small-sample clinical trials, which makes it difficult to provide comprehensive and reliable evidence for clinical practice. Large-scale analyses integrating data across these multiple databases and registries are therefore warranted to clarify the clinical application of tyrosine kinase inhibitors in chronic myeloid leukemia management.

Against this background, this study aimed to characterize the global registration landscape, drug distribution, trial phases, and publication Status of CML-related TKI clinical trials using data from five authoritative public clinical trial databases and registration systems, so as to provide a reference for improving the transparency of clinical trials in the CML field and optimizing the public management of registered trial results.

## 2 Methods

### 2.1 Identification and screening of studies

This registry-based evidence-mapping study was reported in accordance with the PRISMA Extension for Scoping Reviews (PRISMA-ScR), with adaptations appropriate for clinical trial registry data. The completed PRISMA-ScR checklist is provided in Supplementary Table S1. No protocol was registered for this evidence-mapping study.

**FIGURE 1:**
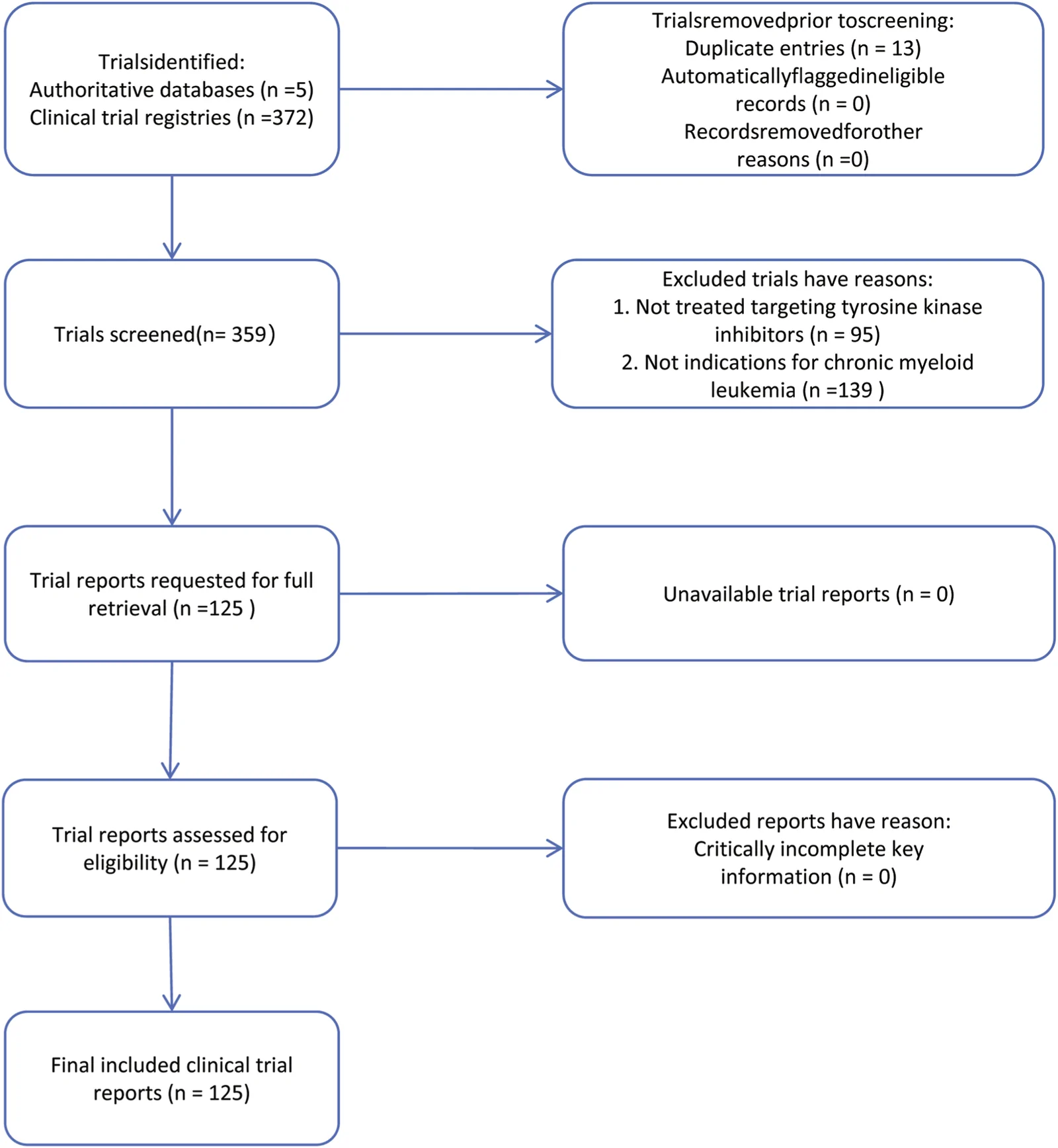
PRISMA flow diagram of trial identification, screening, and inclusion.

We conducted a clinical-trial landscape analysis by searching five clinical-trial registration databases: ClinicalTrials.gov, the China Clinical Trial Registration Center, the ISRCTN Registry, the EU Clinical Trials Register, and the Australian New Zealand Clinical Trials Registry. No restriction was applied to the study start date, and all searches were completed on 19 March 2026. As the search interfaces differed across databases, database-specific search strategies were used. For ClinicalTrials.gov, the search strategy was (Chronic myeloid leukemia OR Leukemia, Granulocytic OR Myeloid Leukemia OR Leukemias, Myeloid OR Myeloid Leukemias OR Leukemia, Myelocytic OR Leukemias, Myelocytic OR Myelocytic Leukemias OR Myelocytic Leukemia OR Leukemia, Myelogenous OR Leukemias, Myelogenous OR Myelogenous Leukemias OR Granulocytic Leukemia OR Granulocytic Leukemias OR Leukemias, Granulocytic OR Myelogenous Leukemia OR Leukemia, Monocytic, Chronic OR Monocytic Leukemia, Chronic OR Chronic Monocytic Leukemia OR Chronic Monocytic Leukemias OR Leukemia, Chronic Monocytic OR Leukemias, Chronic Monocytic OR Monocytic Leukemias, Chronic) AND (Tyrosine kinase inhibitor OR Inhibitors, Tyrosine Kinase OR Kinase Inhibitors, Tyrosine OR Tyrosine Protein Kinase Inhibitors OR Tyrosine Kinase Inhibitor OR TKI Tyrosine Kinase Inhibitors). For the China Clinical Trial Registration Center, “慢性粒细胞白血病” was entered in the research disease name field. For the ISRCTN Registry, “Chronic myeloid leukemia” was entered as a text search term. For the EU Clinical Trials Register, the search strategy was (Tyrosine kinase inhibitor OR Inhibitors, Tyrosine Kinase OR Kinase Inhibitors, Tyrosine OR Tyrosine Protein Kinase Inhibitors OR Tyrosine Kinase Inhibitor OR TKI Tyrosine Kinase Inhibitors). For the Australian New Zealand Clinical Trials Registry, the search strategy was: (CML OR Chronic myelogenous OR Chronic monocytic leukemia) AND (Tyrosine kinase inhibitor OR TKI). Because the search interfaces, indexing systems, and available filters differed across registries, database-specific search strategies were developed and applied. The complete search strategy, including database-specific search terms, fields, and records retrieved, is provided in Appendix 1. All retrieved records were exported to Excel and harmonized before screening. A total of 372 potentially eligible studies were initially identified, and the screening process was performed by investigators Zhang and Tian (Figure 1). The detailed procedures are as follows:

1. Duplicate record identification and reconciliation: Duplicate records were initially identified by matching trial registration numbers. When the same trial was registered in more than one registry under different identifiers, records were assessed using the trial title or acronym, sponsor or principal investigator, participating institutions, intervention and treatment regimen, study design, planned sample size, and study start date. Records judged to represent the same trial were consolidated into a single analytic record. When information differed across registry records, the record with the most complete information, or the primary registration record when identifiable, was retained. Duplicate assessment and reconciliation were independently performed by Zhang and Tian; disagreements were resolved through discussion, with adjudication by Tang when necessary.
2. Preliminary screening was conducted based on titles, interventions and disease types. First, initial screening was performed according to titles, interventions and disease types to exclude studies that obviously did not meet the inclusion criteria. The inclusion criteria are listed as follows: ① The indication was limited solely to chronic myeloid leukemia, or the study participants were required to conform to the National Comprehensive Cancer Network (NCCN) Clinical Practice Guidelines in Oncology: Chronic Myeloid Leukemia (Shah et al., 2024; Deininger et al., 2020; Radich et al., 2018). ② Drug categories were defined *a priori* for the purpose of this study and were not intended to reproduce the terminology used by National Comprehensive Cancer Network (NCCN) or European LeukemiaNet (ELN) guidelines. Core CML TKIs were defined as BCR::ABL1-targeted agents recommended for CML in NCCN or ELN guidance and/or approved for a CML indication in jurisdictions represented by the searched registries. CML-related investigational drugs were BCR::ABL1-targeted agents evaluated in dedicated CML cohorts but without a guideline recommendation or regulatory CML indication at the search date (Shah et al., 2024; Apperley et al., 2025). Non-core targeted drugs were agents with primary targets other than BCR::ABL1 that were evaluated in CML, usually as adjunctive or combination treatments, but had neither a guideline-recommended nor regulatory CML indication; ③ All trials fully meeting the above inclusion criteria were enrolled in the analysis, irrespective of trial status (e.g., completed, recruiting, ongoing but not yet recruiting, unknown status) and whether relevant results had been published. Exclusion criteria were as follows: ① Trials involving multiple hematological malignancies simultaneously from which CML subgroup data could not be separately extracted were excluded; ② Non-pharmacological intervention studies were excluded; ③ Diagnostic, prognostic and biomarker studies irrelevant to TKI treatment were excluded; ④ Pharmacokinetic studies conducted in healthy volunteers were excluded, unless explicitly associated with drug development for CML; ⑤ Duplicate registration records were excluded, and only records with the most complete information or those from the primary registration platform were retained.
3. Full-record eligibility assessment stage: Records that passed the preliminary screening were assessed in detail to verify the intervention, disease population, and completeness of the registry information. The focus is to confirm the specific type of intervention (types and administration regimens of tyrosine kinase inhibitors), the diagnostic purity of enrolled participants (whether subgroups of patients with non-chronic myeloid leukemia are included), and the completeness of study design. Records with incomplete information or undisclosed conditions that did not meet the inclusion criteria were excluded.

During the screening process, two researchers (Zhang and Tian) independently recorded the number of included and excluded trials at each stage, along with the reasons for exclusion. Any discrepancies encountered during the screening were first resolved *via* face-to-face discussion to reach a consensus. If no agreement could be reached after discussion, a third researcher (Tang) would make an adjudication, and the arbitral conclusion was taken as the final screening decision.

Registry records were initially exported from the respective registries and harmonized in an Excel database. Trial status and key registry information were manually verified against the original registry record by two independent reviewers. Automated extraction, where used, served only for initial record retrieval and data organization; final eligibility assessment, duplicate reconciliation, trial-status verification, and data classification were performed manually. Disagreements were resolved by discussion and, when necessary, adjudication by a third reviewer.

### 2.2 Data extraction

The key elements extracted and compared included the year and phase of study initiation, molecular targets, trial type, trial status, drug names and research regions. Target classification was assigned at the drug level according to the best available annotated pharmacological target profile of each agent, rather than trial-specific molecular or biomarker evidence. For multikinase inhibitors, the assigned category represents the principal or commonly annotated target and should not be interpreted as evidence that the observed trial outcomes were mediated by that target alone. Missing items were supplemented by reviewing original registry records and publicly available linked information when available. Data extraction was conducted by two researchers (Zhang and Tian), and any inconsistencies or disputes were resolved through discussion with a third researcher (Tang), who made the final adjudication. No formal critical appraisal of individual registry records was undertaken because this study aimed to map registered trial characteristics and results-disclosure patterns rather than estimate intervention effects. In addition, registry-recorded lead sponsor information and registered enrollment were extracted. Lead sponsors were used as a proxy for funding source and were classified as academic/research-sponsored or industry-sponsored. Academic/research-sponsored trials included universities, hospitals, research institutions, non-profit clinical research organizations, and cooperative research networks. Industry-sponsored trials included pharmaceutical companies, biotechnology companies, other for-profit companies, and contract research organizations. Trial size was categorized according to registered enrollment as <50, 50–99, 100–299, or ≥300 participants. Additional trial-design characteristics extracted from registry records included intervention model, masking, and primary outcome measures. Intervention model and masking were obtained from the registry design fields. Observational studies and trials without usable structured design information were classified as “not reported/not applicable” for these variables. Primary endpoint category was assigned according to the first listed primary outcome measure and its accompanying description, to generate mutually exclusive categories: molecular/cytogenetic/hematologic response, safety/tolerability, survival/time-to-event, treatment-free remission, pharmacokinetic/pharmacodynamic, other, and not reported. Trials without a usable primary outcome measure were classified as not reported.

For the integrated Sankey visualization, all included trials were assigned to one mutually exclusive category within each of four dimensions: study drug generation/class, principal annotated target profile, clinical phase, and registry-recorded trial status. For multi-agent trials, study drug class was assigned using a prespecified hierarchy: allosteric BCR-ABL1 inhibitor, third-generation TKI, investigational BCR-ABL1 inhibitor, second-generation TKI, first-generation TKI, other targeted/combination therapy, other/uncertain drug class, and not reported/not applicable. Principal annotated target profiles were classified as BCR-ABL1 only, BCR-ABL1 plus KIT/PDGFR, BCR-ABL1 plus SRC, BCR-ABL1 plus other kinases, other/non-BCR-ABL1 target, or not reported/not applicable. Clinical phase was categorized as Phase I, Phase I/II, Phase II, Phase III, Phase IV, or not applicable/not reported. Registry-recorded trial status was categorized as completed, active, not yet recruiting, discontinued, or unknown. The Sankey diagram was used for descriptive visualization only.

### 2.3 Assessment of publication status

Publication was assessed among trials with a status of “Completed” and a completion date at least 2 years before the search date. The 2-year interval was selected to reduce the likelihood of classifying recently completed trials as lacking publication before sufficient time had elapsed for data cleaning, analysis, and results submission. This conservative threshold is longer than the standard 1-year results-submission timeline for applicable clinical trials subject to Food and Drug Administration Amendments Act of 2007 (FDAAA) 801 and 42 CFR Part 11 on ClinicalTrials.gov; however, reporting requirements and permissible extensions vary across registries and trial types. Registry records were first assessed using the official trial records. We then conducted a supplementary bibliographic search for each eligible completed trial in PubMed, Embase, and Google Scholar from database inception to 19 March 2026. Searches were performed using the trial registration identifier; the trial title or acronym, intervention name, and lead investigator were additionally used when necessary to identify potentially eligible reports. Full-length peer-reviewed articles reporting trial-specific clinical efficacy, safety, or other outcome data were classified as results publications. Publicly available results were defined as the availability of either registry records or an eligible peer-reviewed results publication. Descriptive analyses were conducted according to trial phase, pharmacological target profile, and individual drug exposure. For drug- and target-level analyses, trials without pharmacological interventions were excluded. For phase-specific analyses, trials with undisclosed phase information were excluded. Prespecified inferential subgroup analyses were conducted according to trial phase and individual drug exposure. Because a trial could involve more than one drug, drug-specific analyses compared trials involving each agent with trials not involving that agent. Among completed trials eligible for publication assessment, publication status was compared according to sponsor type and registered trial size. Sponsor-type comparisons were performed using a two-sided Fisher exact test. Trial-size comparisons were performed using the Fisher–Freeman–Halton exact test. Trials with unavailable enrollment data were excluded from the trial-size comparison. All analyses were exploratory and univariable. Country-level disclosure comparisons were not performed because trials could be multinational and several country-specific strata had sparse numbers of eligible completed trials; geographic findings were therefore presented descriptively.

### 2.4 Statistical analysis

Statistical analyses were primarily descriptive. Categorical variables are presented as frequencies and percentages. Publication rates were calculated overall and within prespecified subgroups among eligible completed trials, with exact binomial 95% confidence intervals. Between-group differences in disclosure rates were assessed using two-sided Fisher’s exact tests; for comparisons involving more than two trial-phase categories, the Fisher–Freeman–Halton exact test was used. P values from drug-specific analyses were adjusted for multiple comparisons using the Benjamini–Hochberg procedure. All analyses were performed using R software (version 4.5.3), and a two-sided P value <0.05 was considered statistically significant. Multivariable analyses were restricted to complete cases with available sponsor type, trial enrollment, and classifiable trial phase. Firth penalized logistic regression was used to reduce small-sample bias and address sparse outcome cells. For the country-level contextual analysis, only trials with a recorded country role (single-country trial, leading country, or participating country) were included. Each country was counted no more than once per trial; however, a multinational trial could contribute to more than one country. Country-specific trial-activity density was calculated as the number of country-trial involvements per 10 million population, using 2023 population estimates from the United Nations World Population Prospects 2024 Revision (United Nations and D.O.E.A., 2024). The normalized indicators were considered descriptive and were not interpreted as measures of national research productivity.

## 3 Results

A total of 125 trials ultimately met the inclusion criteria (Appendix 2). Inter-rater reliability regarding binary inclusion and exclusion judgments for 359 trials was evaluated by two independent reviewers (Zhang and Tian). The observed agreement reached 93.59% (336/359), with a Cohen’s Kappa coefficient of 0.86. Statistical analysis showed that apart from one prognostic study (ChiCTR-PRNRC-09000307), there were 96 interventional studies (96/125, 76.8%) and 28 observational studies (28/125, 22.4%). One prognostic study (ChiCTR-PRNRC-09000307) was included. It investigated the role of post transplant BCR/ABL transcript levels in guiding pre-emptive clinical management after HLA-matched hematopoietic stem-cell transplantation; imatinib was listed as one of the management approaches. The study retained its registry classification as a prognostic study and was included only in the overall descriptive landscape analysis. Observational studies were included because they constituted part of the registered research activity related to TKI use in chronic myeloid leukemia. Their inclusion enabled a more comprehensive description of the distribution of study designs in this research field.

Approximately one-fifth of the included studies were collaborative trials (21.6%). Trial NCT04971226 involved the greatest number of participating countries, spanning 28 countries and 110 research centers. Nearly two-thirds of the available evidence originated from single-center investigations. Further multicenter collaborative research would help enrich the clinical evidence base. Additional trial characteristics are summarized in Table 1. Overall, 23 trials (18.4%) were randomized, 74 (59.2%) were non-randomized, and 28 (22.4%) had unreported or non-applicable allocation. Regarding intervention model, 57 trials (45.6%) used a single-group assignment, 23 (18.4%) used a parallel assignment, 1 (0.8%) used a crossover assignment, and 4 (3.2%) used a sequential assignment; intervention model was not reported or was not applicable for 40 trials (32.0%). Eighty-five trials (68.0%) were registered as open-label, whereas masking information was not reported or was not applicable for the remaining 40 trials (32.0%). Most trials enrolled adults only (108/125, 86.4%); 2 trials (1.6%) enrolled pediatric/adolescent populations, 8 (6.4%) enrolled mixed-age populations, and 7 (5.6%) did not report age eligibility. Ten trials (8.0%) were conducted in the first-line setting and 56 (44.8%) in the later-line setting, whereas 59 (47.2%) were classified as mixed/unspecified according to registry information. Based on the first listed primary outcome, molecular/cytogenetic/hematologic response was the most common primary endpoint category (55/125, 44.0%), followed by treatment-free remission (20/125, 16.0%) and safety/tolerability (19/125, 15.2%). Pharmacokinetic/pharmacodynamic outcomes accounted for six trials (4.8%), survival/time-to-event outcomes for 5 trials (4.0%), and other outcomes for 5 trials (4.0%); the primary endpoint was not reported for 15 trials (12.0%). Registered enrollment was fewer than 50 participants in 41 trials (32.8%), 50–99 in 19 (15.2%), 100–299 in 34 (27.2%), and ≥300 in 13 (10.4%); enrollment was not reported for 18 trials (14.4%). Using the registry-recorded lead sponsor as a funding-source proxy, 63 trials (50.4%) were academic/research-sponsored, 47 (37.6%) were industry-sponsored, and sponsor information was unavailable or unclassifiable for 15 trials (12.0%).

**TABLE 1:** Characteristics of included clinical trials (N = 125).

Characteristic	Category	No. of cases (n/N)	Proportion (%)
Trial type (N = 125)	Interventional trial	96/125	76.8
​	Observational trial	28/125	22.4
​	Prognostic study	1/125	0.8
Trial phase (N = 94; excluding 25 trials with undisclosed phases and 6 without clearly defined targets)	Phase I	8/94	8.5
​	Phase I/II	7/94	7.5
​	Phase II	54/94	57.4
​	Phase III	16/94	17.0
​	Phase IV	5/94	5.3
​	N/A	4/94	4.3
Trial status	Completed	57/125	45.6
​	Recruiting	14/125	11.2
​	Active, not recruiting	16/125	12.8
​	Termination	12/125	9.6
​	Unknown	12/125	9.6
​	Not yet recruiting	10/125	8.0
​	Others	4/125	3.2
Allocation	Randomized	23/125	18.4
​	Non-randomized	74/125	59.2
​	Not reported/not applicable	28/125	22.4
Study population	Adult	108/125	86.4
​	Pediatric/adolescent	2/125	1.6
​	Mixed-age	8/125	6.4
​	Not reported	7/125	5.6
Treatment setting	First-line	10/125	8.0
​	Later-line	56/125	44.8
​	Mixed/unspecified	59/125	47.2
Sample size distribution	<50 participants	41/125	32.8
​	50–99 participants	19/125	15.2
​	100–299 participants	34/125	27.2
​	≥300 participants	13/125	10.4
​	Not reported	18/125	14.4
Sponsor type	Academic/research-sponsored	63/125	50.4
​	Industry-sponsored	47/125	37.6
​	Not reported/unclassifiable	15/125	12.0
Intervention model	Single-group assignment	57/125	45.6
​	Parallel assignment	23/125	18.4
​	Crossover assignment	1/125	0.8
​	Sequential assignment	4/125	3.2
​	Not reported/not applicable	40/125	32.0
Masking	Open-label (no masking)	85/125	68.0
​	Not reported/not applicable	40/125	32.0
Primary endpoint category	Molecular/cytogenetic/hematologic response	55/125	44.0
​	Safety/tolerability	19/125	15.2
​	Survival/time-to-event	5/125	4.0
​	Treatment-free remission	20/125	16.0
​	Pharmacokinetic/pharmacodynamic	6/125	4.8
​	Other	5/125	4.0
​	Not reported	15/125	12.0

The “Others” category of trial status includes interim trial halt, targeted enrollment invitation, temporary trial suspension, and registration withdrawal. Data were extracted from trial registry records using prespecified classification rules. For allocation, single-group interventional studies with allocation recorded as “not applicable” were classified as non-randomized; trials without usable allocation information or non-interventional/observational studies were classified as not reported/not applicable. Adult trials included studies enrolling adults only; mixed-age trials included both pediatric/adolescent and adult participants. Treatment setting was classified as first-line when the registry explicitly described newly diagnosed, untreated, or first-line CML; and as later-line when it explicitly described prior TKI, exposure, resistance, intolerance, treatment failure, relapse, refractory disease, or second-/third-line treatment. “Mixed/unspecified” indicates that the registry record included both first-line and later-line contexts, or did not provide sufficient information to assign a single treatment setting reliably; it does not necessarily mean that the study enrolled a mixed-line population. Registered enrollment was used to classify trial size; actual enrollment was used when available, otherwise anticipated enrollment was used. Sponsor type was classified using the registry-recorded lead sponsor as a proxy for funding source rather than as confirmation of the ultimate funder. Academic/research-sponsored trials included universities, hospitals, medical centers, research institutes, nonprofit research organizations, and collaborative research networks. Industry-sponsored trials included trials with an industry lead sponsor; GWT-TUD GmbH was classified as industry-sponsored. Fifteen trials registered outside ClinicalTrials.gov lacked sufficiently verifiable lead-sponsor information and were classified as not reported/unclassifiable. Intervention model and masking were extracted from registry design fields. Primary endpoint categories were assigned according to the first listed primary outcome measure. “Not reported/not applicable” includes observational studies and trials without usable structured registry information.

### 3.1 International collaboration and demographic characteristics

**FIGURE 2:**
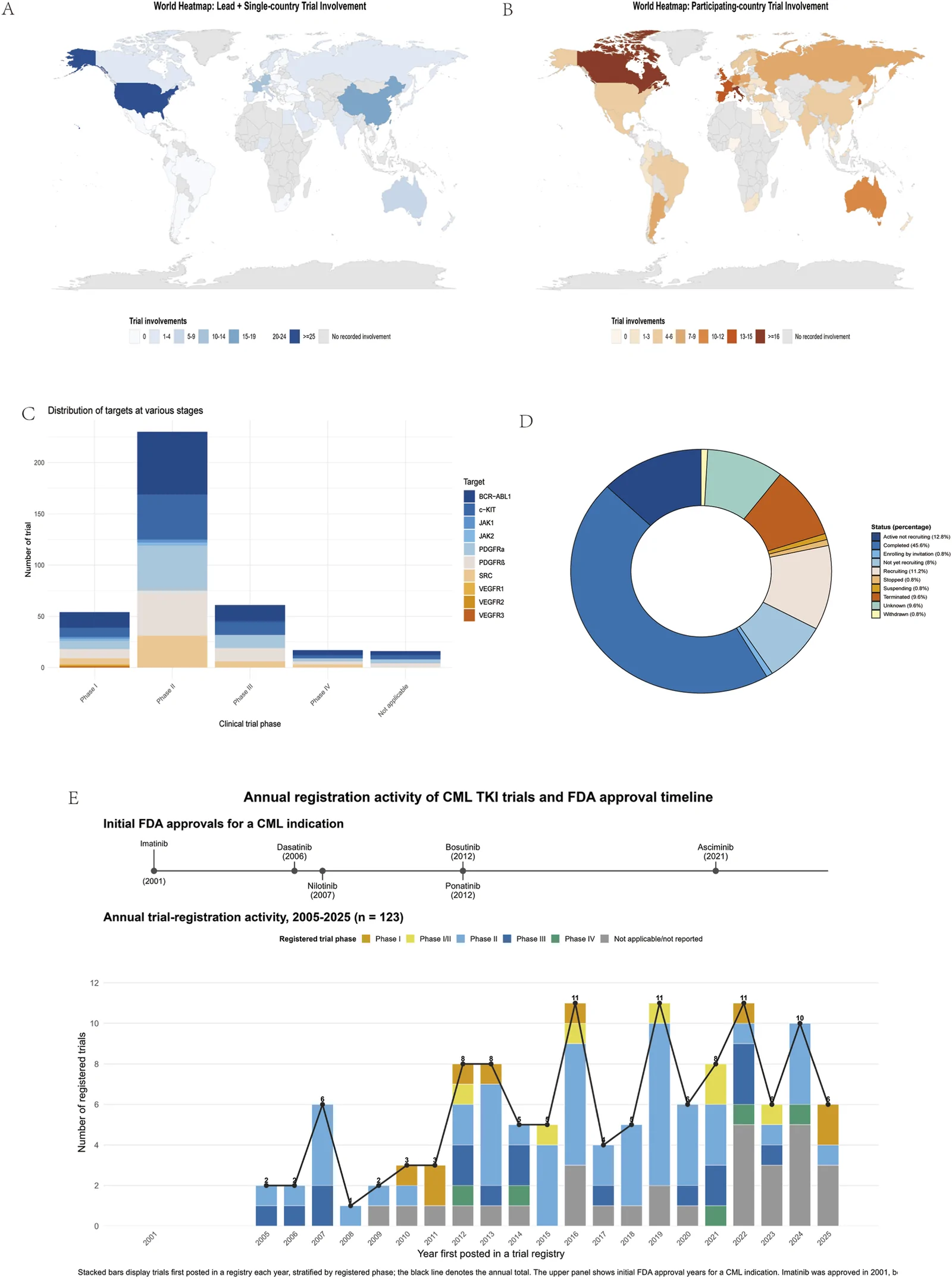
Global distribution and overall characteristics of registered CML TKI trials. **(A)** Leading-country and single-country trial involvement. **(B)** Participating-country trial involvement. **(C)** Distribution of annotated drug target profiles across registered clinical phases. **(D)** Registry-recorded trial status among 125 included trials. **(E)** Annual trial-registration activity and initial FDA approval timeline for CML TKIs.

Among the 125 included trials, country-role data were available for 111 trials (88.8%). The remaining 14 trials had no country assignment in the extracted registry records and could not be classified as single-country, leading-country, or participating-country trials; they were therefore excluded from the country-level analysis only. Country-level trial activity was summarized according to three roles: single-country trials, leading-country trials, and participating-country trials. Each country was counted no more than once per trial, although multinational trials could contribute to more than one country (Figures 2A,B).

By absolute country-trial involvement, the United States was the most frequently represented country, with 38 trials, including 20 single-country trials, 12 leading-country trials, and six participating-country trials. Italy ranked second with 27 trial involvements, comprising 9 single-country trials and 18 participating-country trials. France ranked third with 26 trial involvements, including 8 single-country trials, 3 leading-country trials, and 15 participating-country trials. China ranked fourth, with 23 trial involvements, comprising 17 single-country trials, 1 leading-country trial, and 5 participating-country trials. Germany ranked fifth, with 21 trial involvements, including 3 single-country trials, 7 leading-country trials, and 11 participating-country trials.

Australia and South Korea each had 19 trial involvements; Australia contributed 7 single-country trials and participated in 12 multinational trials, whereas South Korea contributed 3 single-country trials, led 1 trial, and participated in 15 multinational trials. Canada and Spain each had 17 trial involvements. Canada had 1 single-country trial and participated in 16 multinational trials, whereas Spain had 1 single-country trial, led 3 trials, and participated in 13 multinational trials. The United Kingdom had 16 trial involvements, including 3 single-country trials and 13 participating-country trials. Other countries with relatively frequent involvement included the Netherlands (10), Poland (9), Russia (9), Hungary (9), Czechia (9), Argentina (8), Singapore (8), Sweden (7), India (7), Austria (6), Brazil (6), Japan (6), Norway (6), Denmark (5), Finland (5), Belgium (5), Greece (5), Israel (5), Mexico (5), Switzerland (5), and Turkey (5).

After standardization by 2023 population size, the ordering differed substantially from that based on absolute country-trial involvement. For example, the United States and China had 1.1 and 0.2 trial involvements per 10 million population, respectively, despite ranking first and fourth by absolute counts. Among countries/territories with at least five trial involvements, the highest trial-activity densities per 10 million population were observed in Singapore (13.8), Norway (10.9), Hungary (9.3), Finland (8.9), Denmark (8.4), and Czechia (8.3), followed by Australia (7.2), Sweden (6.6), Austria (6.6), and Switzerland (5.6) (Supplementary Table S2). These indicators describe geographical patterns of registered trial activity. Because multinational trials can contribute to multiple countries, they should not be interpreted as direct measures of national research productivity, clinical need, or treatment access.

### 3.2 Assessment of trial publication status

**FIGURE 3:**
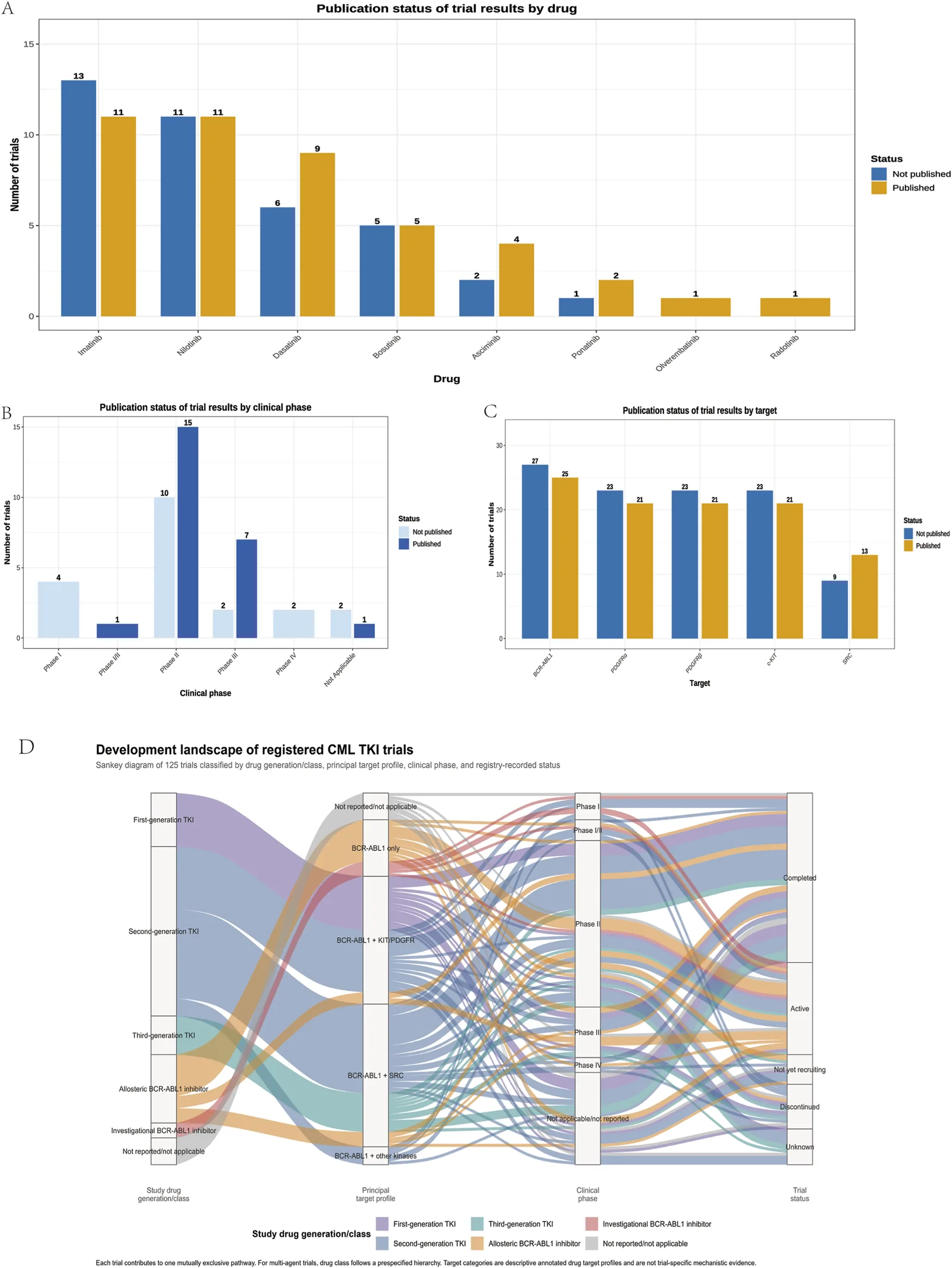
Publication status of trial results and integrated development landscape of registered CML TKI trials. **(A)** Publication status of trial results by drug. **(B)** Publication status of trial results by clinical phase. **(C)** Publication status of trial results by annotated drug target profile. **(D)** Sankey diagram linking study drug generation/class, principal annotated target profile, clinical phase, and registry-recorded trial status.

Publication was assessed among registered studies with a status of completed and a completion date more than 2 years before the search date. A total of 55 studies met these criteria. Of these, three studies did not involve pharmacological interventions and were excluded from the drug- and target-analyses. Therefore, 52 studies with pharmacological interventions were included in the analyses (Figures 3A–C). Among the 52 trials, with an overall publication rate of 48.1% (25/52; 95% CI: 34.0%–62.3%). BCR-ABL1 was the disease-defining therapeutic target across the included CML trials. Several agents also had annotated activity against other kinases, including PDGFRα/β, c-KIT, and SRC. Therefore, target-level analyses should be interpreted as drug target-profile analyses rather than direct evidence that these targets were trial-specific therapeutic mechanisms. Among trials involving agents annotated with PDGFRα, PDGFRβ, or c-KIT as known targets, the publication rate was 47.7% (21/44; 95% CI: 32.5%–63.3%). Trials involving agents annotated with SRC had a disclosure rate of 59.1% (13/22; 95% CI: 36.4%–79.3%). Overall, publication were identified for fewer than half of the pharmacological trials. Disclosure rates varied descriptively across drug target-profile groups; however, these groups were not mutually exclusive because individual agents could have multiple annotated targets. Therefore, these findings should be interpreted as descriptive evidence of incomplete publication rather than as evidence of target-specific differences in reporting practices.

Among 55 trials, 11 did not report clinical phase information and were excluded from the phase-specific results-disclosure analysis. Among the 44 trials with reported clinical phase information, results were disclosed for 24 trials (54.5%) and were not disclosed for 20 trials. No results were disclosed for any Phase I trial (0/4) or Phase IV trial (0/2). The only Phase I/II trial had disclosed results (1/1). Phase II trials represented the largest subgroup (n = 25); results were disclosed for 15 trials and were not disclosed for 10 trials, yielding a disclosure rate of 60.0% (95% CI: 38.7%–78.9%). Phase III trials had the highest disclosure rate, with results disclosed for seven of nine trials and not disclosed for two trials (77.8%; 95% CI: 40.0%–97.2%). Among trials classified as not applicable, results were disclosed for one of three trials (33.3%), while two remained undisclosed. Overall, publication rates differed across clinical phases (Fisher–Freeman–Halton exact test, P = 0.027; Table 2). However, this association should be interpreted cautiously because the Phase I/II, Phase IV, and not-applicable categories contained only one to three trials.

**TABLE 2:** Public results availability by trial phase.

Trial phase	Trials with publicly available results, n/N	Disclosure rate, %	Overall *P* value
Phase I	0/4	0.0	0.027
Phase I/II	1/1	100.0	​
Phase II	15/25	60.0	​
Phase III	7/9	77.8	​
Phase IV	0/2	0.0	​
N/A	1/3	33.3	​
Total	24/44	54.5	​

Eleven trials with undisclosed phase information were excluded. P value was calculated using the Fisher–Freeman–Halton exact test.

Within this pharmacological subset, drug-specific subgroups were not mutually exclusive because individual trials could involve more than one agent. Publication rates varied descriptively across individual agents, ranging from 45.8% for imatinib (11/24; 95% CI: 25.6%–67.2%) to 66.7% for asciminib (4/6; 95% CI: 22.3%–95.7%) and ponatinib (2/3; 95% CI: 9.4%–99.2%). The corresponding rates were 50.0% for nilotinib (11/22; 95% CI: 28.2%–71.8%), 60.0% for dasatinib (9/15; 95% CI: 32.3%–83.7%), and 50.0% for bosutinib (5/10; 95% CI: 18.7%–81.3%). Results were disclosed for the single olverembatinib trial and the single radotinib trial (100.0% each); however, these estimates were based on only one trial per drug. In comparisons of trials involving each agent with trials not involving that agent, no statistically significant differences in publication rates were observed in unadjusted Fisher’s exact tests (all unadjusted P ≥ 0.362) or after Benjamini–Hochberg adjustment (all adjusted P = 1.000; Table 3).

**TABLE 3:** Public results availability by individual drug exposure.

Drug	Trials involving the drug, disclosed n/N (%)	Trials not involving the drug, disclosed n/N (%)	Unadjusted *P* value	BH-adjusted *P* value
Imatinib*	11/24 (45.8)	14/28 (50.0)	0.788	1.000
Nilotinib	11/22 (50.0)	14/30 (46.7)	1.000	1.000
Dasatinib	9/15 (60.0)	16/37 (43.2)	0.362	1.000
Bosutinib	5/10 (50.0)	20/42 (47.6)	1.000	1.000
Ponatinib	2/3 (66.7)	23/49 (46.9)	0.603	1.000
Asciminib	4/6 (66.7)	21/46 (45.7)	0.411	1.000
Olverembatinib	1/1 (100.0)	24/51 (47.1)	0.481	1.000
Radotinib	1/1 (100.0)	24/51 (47.1)	0.481	1.000

Each drug was analyzed separately; trials involving multiple drugs could contribute to more than one drug-specific comparison. P values were calculated using two-sided Fisher’s exact tests and adjusted using the Benjamini–Hochberg procedure.

Among 55 completed trials eligible for publication assessment, 25 (45.5%; 95% CI, 33.0%–58.5%) had an identified publication. Publication rates did not differ by sponsor type: 13 of 29 academic/research-sponsored trials (44.8%; 95% CI, 28.4%–62.5%) and 12 of 26 industry-sponsored trials (46.2%; 95% CI, 28.8%–64.5%) had an identified publication (P = 1.000). Among the 54 trials with available registered enrollment data, publication rates were 26.7% (4/15; 95% CI, 10.9%–52.0%) for trials enrolling fewer than 50 participants, 40.0% (4/10; 95% CI, 16.8%–68.7%) for those enrolling 50–99 participants, 63.2% (12/19; 95% CI, 41.0%–80.9%) for those enrolling 100–299 participants, and 40.0% (4/10; 95% CI, 16.8%–68.7%) for those enrolling at least 300 participants. Although trials enrolling 100–299 participants had the highest observed publication rate, the overall difference across trial-size categories was not statistically significant (P = 0.191; Table 4). One trial with unreported enrollment was excluded from the trial-size comparison.

**TABLE 4:** Association of sponsor type and trial size with publication status. Completed trials eligible for publication assessment (N = 55).

Characteristic	Category	Published, n/N (%)	Not published, n/N (%)	*P* value
Sponsor type (funding-source proxy)	Academic/research-sponsored	13/29 (44.8%)	16/29 (55.2%)	1.000
​	Industry-sponsored	12/26 (46.2%)	14/26 (53.8%)	​
Registered trial size	<50 participants	4/15 (26.7%)	11/15 (73.3%)	0.191
​	50–99 participants	4/10 (40.0%)	6/10 (60.0%)	​
​	100–299 participants	12/19 (63.2%)	7/19 (36.8%)	​
​	≥300 participants	4/10 (40.0%)	6/10 (60.0%)	​
​	Not reported (excluded from test)	1/1 (100.0%)	0/1 (0.0%)	​

Funding source was represented by the registry-recorded lead sponsor. Sponsor-type comparison used a two-sided Fisher exact test. Trial-size comparison used the Fisher–Freeman–Halton exact test and excluded trials with unreported enrollment.

Multivariable analysis of factors associated with publication. Among 44 completed trials with evaluable results-disclosure status and registry-recorded phase information, 40 were included in the complete-case multivariable model. Of the 44 trials, three had a phase recorded as not applicable and therefore could not be categorized as early phase (Phase I, Phase I/II, or Phase II) or late phase (Phase III or Phase IV), and one had unavailable trial-enrollment data; these four trials were excluded from the complete-case analysis. Firth penalized logistic regression was used because of the modest sample size and sparse outcome cells in individual phase categories, which could lead to unstable estimates in conventional logistic regression. Among the 40 included trials, 22 (55.0%) had an identified publication. After adjustment for sponsor type, log_2_-transformed trial enrollment, and trial phase, industry sponsorship was not significantly associated with publication compared with academic/research sponsorship (adjusted odds ratio [aOR], 1.37; 95% confidence interval [CI], 0.35–5.37; P = 0.655). Each doubling of trial enrollment was associated with higher odds of publication (aOR, 1.66; 95% CI, 1.04–2.65; P = 0.033). Late-phase trials were not significantly associated with publication compared with early-phase trials (aOR, 0.69; 95% CI, 0.13–3.52; P = 0.654). These findings should be interpreted as exploratory because of the limited sample size and the small number of outcome events in some subgroups (Table 5).

**TABLE 5:** Multivariable Firth penalized logistic regression of publication. Complete-case analytic sample: N = 40; results disclosed: 22/40 (55.0%). Firth model converged in 10 iterations.

Predictor	Adjusted OR	95% CI	Wald SE	P value	Reference / unit
Industry-sponsored trials (reference: academic/research-sponsored trials)	1.37	0.35–5.37	0.698	0.655	Reference stated in predictor
Trial enrollment (per doubling)	1.66	1.04–2.65	0.238	0.033	Per doubling
Late-phase trials (Phase III/IV) (reference: early-phase trials [Phase I, Phase I/II, or Phase II])	0.69	0.13–3.52	0.833	0.654	Reference stated in predictor

Publication status was determined through manual searches of registry records and bibliographic databases. A trial was classified as published when an eligible full-length peer-reviewed article reporting trial-specific clinical efficacy, safety, or other outcome data was identified, as described in Section 2.3. The multivariable Firth penalized logistic regression model included 40 completed trials with complete data on sponsor type, trial enrollment, and classifiable clinical phase. Four of 44 trials were excluded because of a non-classifiable phase (n = 3) or unavailable enrollment data (n = 1). Trial enrollment was log_2_-transformed; the corresponding aOR represents the association per doubling of enrollment. Early phase included Phase I, Phase I/II, and Phase II; late phase included Phase III and Phase IV. Academic/research-sponsored trials and early-phase trials were the reference categories.

aOR, adjusted odds ratio; CI, confidence interval.

### 3.3 Stage and objective analysis

Stratified distribution analysis of trial quantities for each therapeutic target across different clinical trial phases (Figure 2C).

Among the 125 clinical trials included in the analysis, 25 were excluded because their trial phases were undisclosed, and six were further excluded because no clearly defined target was reported. Therefore, the analysis was based on 94 eligible clinical trials, which showed a clear phase-distribution pattern: the number of studies increased from Phase I and peaked at Phase II, followed by a gradual decrease in the number of Phase III and Phase IV trials with the advancement of clinical development. Phase II trials accounted for the largest proportion (n = 54), followed by Phase III trials (n = 16). There were 8 Phase I trials, 7 Phase I/II trials and 5 Phase IV trials. In addition, 4 trials were not assigned to any specific trial phase (not applicable).

When stratified by pharmacological target profile, BCR-ABL1-targeted agents accounted for the majority of trials across clinical development phases. Specifically, there were 52 BCR-ABL1-targeted trials in Phase II, 16 in Phase III, 8 in Phase I, 5 in Phase IV, and 7 in Phase I/II. Agents with annotated activity against SRC, PDGFRα/β, or c-KIT were mainly represented in early- and mid-phase studies. Less frequently investigated non-BCR-ABL1 target pathways, such as JAK- and VEGFR-related pathways, were observed only in sporadic early-phase exploratory trials. These findings indicate that registered trial activity was concentrated on BCR-ABL1-targeted agents and Phase II studies, whereas non-BCR-ABL1 target profiles were less frequently represented in later-phase clinical trials.

### 3.4 Temporal distribution of registered trial activity by phase

Although 125 trials met the overall inclusion criteria, two trials were first posted in 2026. Because 2026 was an incomplete calendar year at the time of the search, these two records were excluded from the temporal analysis to avoid comparing incomplete annual registration activity with complete years. Therefore, 123 trials with a registry-recorded First Posted date between 2005 and 2025 were included in the annual time-trend analysis (Figure 2E). Annual trial-registration activity varied over time. The number of trials first posted in registries was low during 2005–2011, ranging from 1 to 6 trials per year. Registration activity increased thereafter, with 8 trials recorded in both 2012 and 2013, followed by fluctuations in subsequent years. The highest annual total was 11 trials, observed in 2016, 2019, and 2022; an additional increase was observed in 2024 (10 trials). Overall, the temporal pattern showed intermittent peaks and year-to-year variation rather than a sustained monotonic increase or decline. Phase II was the most frequently recorded phase overall (55/123, 44.7%) and constituted a substantial component of annual registration activity in many years. However, the phase distribution varied across calendar years, and not-applicable/not-reported phase classifications were relatively common in several recent years, including 2022 and 2024. Phase III trials were recorded intermittently throughout the observation period, whereas Phase I, Phase I/II, and Phase IV trials occurred less frequently. The FDA approval dates displayed in the upper panel of Figure 2E are provided as descriptive regulatory context only; no formal association between approval timing and annual trial-registration activity was assessed. In summary, the registered CML TKI trial landscape was characterized by fluctuating annual activity, with recurrent periods of increased registration rather than a consistent long-term decline. Phase II trials accounted for the largest share of registered activity, while early-phase, confirmatory Phase III, and post-marketing Phase IV trials were less commonly recorded.

### 3.5 Analysis of trial status

Of the 125 trials, completed trials accounted for the largest proportion (57/125, 45.6%) (Figure 2D). Trials with the status of ongoing but not recruiting accounted for 12.8%, and those actively recruiting accounted for 11.2%. Both early-terminated trials and trials with unknown status accounted for 9.6%, followed by trials not yet initiating recruitment at 8%. Trials classified as suspended midway through the trial, invitation-only enrollment, temporarily suspended, and withdrawn registration each accounted for 0.8%. This distribution indicates that nearly half of the registered trials had reached completed status, whereas a proportion were terminated or had unknown status.

### 3.6 Integrated development landscape

An integrated overview of the registered development landscape is presented in the Sankey diagram (Figure 3D). Among the 125 included trials, second-generation TKIs constituted the largest drug-class category (57/125, 45.6%), followed by allosteric BCR-ABL1 inhibitors (23/125, 18.4%), first-generation TKIs (18/125, 14.4%), and third-generation TKIs (13/125, 10.4%). The most frequent annotated target profiles were BCR-ABL1 plus SRC (48/125, 38.4%) and BCR-ABL1 plus KIT/PDGFR (43/125, 34.4%). Phase II was the most commonly recorded clinical phase (56/125, 44.8%), and completed trials represented the largest registry-status category (57/125, 45.6%). Each trial was assigned to one mutually exclusive pathway in the Sankey diagram. For multi-agent trials, drug class was assigned according to a prespecified hierarchy, and target categories represent descriptive annotated drug target profiles rather than trial-specific mechanisms.

## 4 Discussion

This study systematically analyzed the current status of clinical research on tyrosine kinase inhibitors for the treatment of chronic myeloid leukemia based on five authoritative clinical trial databases and registration systems. A total of 125 trials meeting the inclusion criteria were finally included, and several key research outcomes were derived.

First, registered CML TKI trials showed geographical heterogeneity in both trial volume and patterns of international participation. In the present registry-based dataset, China was represented predominantly by single-country trials, with fewer recorded leading-country and participating-country roles in multinational studies. This observation should be interpreted cautiously because registry selection, incomplete coverage of regional or domestic registries, language-related differences in record retrieval, and variation in the reporting of multinational trial locations may influence the observed pattern. Similarly, differences in absolute trial counts should not be interpreted solely as differences in research capacity, as registered trial activity may also be shaped by population size, CML burden, drug availability, regulatory requirements, sponsorship strategies, registry coverage, healthcare-system characteristics, and patient-recruitment networks. Population-standardized analyses further showed that country rankings differed from those based on absolute trial counts alone. However, these normalized indicators remain descriptive because multinational trials can contribute to more than one country and because they do not account for cross-country differences in disease burden, healthcare resources, research expenditure, approval timelines, or trial-registration practices. Therefore, the observed geographical patterns describe the distribution of registered trial activity rather than definitive differences in national research productivity, clinical need, or treatment access. Strengthening multicenter collaboration, improving registry completeness, and standardizing endpoint definitions may improve the international comparability and visibility of future CML research.

Second, publicly identifiable results were available for fewer than half of eligible completed pharmacological trials. This finding should not be interpreted as evidence that trials without registry records were never published, because registry review was supplemented by a bibliographic search. Conversely, some eligible publications may not have been identified because of incomplete indexing, absent or inaccurate trial identifiers, inaccessible full texts, or insufficient information to verify linkage with a registry record. The observed estimates should therefore be interpreted as indicators of publicly identifiable results availability rather than definitive evidence of publication status bias or proof that no publication exists. Although publication availability varied across clinical phases, this finding requires cautious interpretation because several phase-specific subgroups were small. Similarly, no drug-specific difference remained statistically significant after adjustment for multiple comparisons. In exploratory analyses, larger trial enrollment was associated with a higher likelihood of publication, whereas sponsor type and dichotomized trial phase were not independently associated with publication. Larger studies may have greater organizational capacity and incentives for dissemination; however, this association was based on a limited complete-case sample and should be regarded as hypothesis-generating rather than causal evidence. Incomplete public availability of trial results may reduce the completeness of evidence available for evidence synthesis, guideline development, and future trial planning (DeVito et al., 2020).

Incomplete public availability of trial results also has important clinical implications. For evidence synthesis, unavailable or difficult-to-identify results may lead to an incomplete representation of the available evidence and increase uncertainty in pooled estimates of efficacy and safety. For clinical guideline development, gaps in the accessible evidence base may limit the ability of guideline panels to assess the consistency, applicability, and certainty of evidence supporting treatment recommendations. In health technology assessment, incomplete reporting may complicate evaluations of comparative effectiveness, long-term safety, treatment value, and resource allocation. Regulatory decision-making may likewise be affected when post-marketing, safety, or confirmatory trial findings are not readily accessible for independent assessment. At the patient-care level, incomplete evidence availability may hinder informed shared decision-making, particularly when clinicians and patients need to weigh the benefits and risks of different TKIs in the context of resistance, intolerance, comorbidities, and long-term treatment goals. These implications reinforce the importance of timely and complete reporting of completed CML TKI trials.

These findings should also be considered in the context of previous oncology clinical-trial transparency research. In a large cohort of 12,240 interventional oncology trials registered on ClinicalTrials.gov, Liu et al. reported that 60.7% had results available in a registry or journal publication within 24 months of primary completion, and larger trials were more likely to report results (Liu et al., 2021). The proportion of publicly identifiable results in our CML-specific dataset was lower (48.1%, 25/52); however, direct numerical comparisons should be interpreted cautiously because the studies differed in disease scope, registry coverage, eligibility criteria, and follow-up definitions. Nevertheless, both studies suggest that incomplete public availability of trial results remains a relevant concern in oncology and that trial size may influence the dissemination of trial findings. In addition, In a previous comparison of ClinicalTrials.gov records and corresponding publications for Phase III/IV randomized cancer-drug trials, registry records contained more complete reporting of serious and other adverse events than journal articles (Lv et al., 2017). Together, these findings support the use of both registry records and bibliographic searches when evaluating the evidence base for CML therapies and reinforce the importance of complete reporting across both sources.

Third, the predominance of Phase II trials may reflect the contemporary development pathway for CML therapies. The availability of highly effective standard TKIs and the relatively indolent course of chronic-phase CML make large definitive trials difficult, resource-intensive, and dependent on prolonged follow-up. For novel agents, combination regimens, dose-optimization approaches, and treatments for TKI-resistant or TKI-intolerant disease, Phase II studies provide a practical means of evaluating preliminary molecular efficacy, safety, and tolerability before proceeding to large comparative trials. In contrast, Phase III studies generally require comparisons with effective established therapies, larger sample sizes, and longer follow-up to demonstrate clinically meaningful advantages in durable molecular response, treatment-free remission, progression, or survival. Therefore, the observed Phase II predominance may represent a pragmatic balance between proof-of-concept evaluation and the logistical challenges of confirmatory development. This interpretation remains exploratory because registry records do not capture sponsors’ development strategies or the reasons why individual programs did not progress to later-phase trials. The concentration of registered activity on BCR-ABL1-directed therapies is consistent with the central biological role of BCR-ABL1 in CML and the mature development pathway for BCR-ABL1-targeted agents. Less frequently investigated non-BCR-ABL1 pathways may represent areas for further exploratory research, but target categories should be interpreted as descriptive drug pharmacology rather than trial-specific mechanisms (Amarante-Mendes et al., 2022). Although Phase II predominance is not itself evidence of an inadequate development pathway, the limited representation of Phase I, Phase III, and Phase IV trials may indicate potential gaps in early proof-of-concept development, confirmatory comparative evaluation, and post-marketing evidence generation (Braun et al., 2020; Yang et al., 2024).

Future CML TKI research should address refined clinical priorities beyond survival, including deep molecular response, treatment-free remission (TFR), TKI resistance and intolerance, long-term toxicities, cardiovascular and hepatic safety, and treatment in special populations (Shah et al., 2024). Registry-based evidence mapping is useful for identifying whether these topics have entered the clinical development pipeline, but it cannot replace systematic reviews, individual-patient-data analyses, comparative effectiveness studies, or real-world evidence. The present findings are therefore most relevant to the research landscape and transparency rather than to comparisons of the relative clinical merits of individual TKIs.

Recent therapeutic developments should be considered within this evolving landscape. Asciminib, an allosteric inhibitor targeting the ABL myristoyl pocket, has demonstrated favourable efficacy and safety as a first-line treatment strategy for adults with newly diagnosed Philadelphia chromosome-positive CML in chronic phase in the phase III ASC4FIRST trial (Apperley et al., 2025; Hochhaus et al., 2024). TFR has also become an important patient-centred therapeutic goal, requiring sustained deep molecular response, appropriate patient selection, and reliable molecular monitoring. In addition to asciminib, further studies of novel allosteric inhibitors and rational combination strategies may help address resistance, intolerance, and advanced-phase disease. Future precision-medicine approaches should integrate BCR::ABL1 mutation status, disease risk, molecular response kinetics, comorbidities, treatment tolerance, and patient preferences to guide TKI selection and dosing. Registries should capture these clinically relevant features more consistently to facilitate assessment of how emerging therapies are incorporated into CML care.

Specific actions are needed from the main stakeholders in CML clinical research. Investigators should prospectively register trials, provide complete structured information on study design, eligibility criteria, molecular endpoints, and treatment setting, update trial status promptly, and include the registration identifier in all resulting publications. Sponsors should support adequately designed multicentre studies, ensure timely submission of results regardless of study findings, and facilitate transparent reporting of long-term efficacy and safety outcomes. Regulatory agencies and research ethics committees should strengthen monitoring of registration and results-reporting compliance, particularly for completed, terminated, and post-marketing studies. Registry administrators should improve the completeness and interoperability of registry records by requiring standardized fields for sponsor role, enrollment, trial phase, molecular characteristics, treatment line, and links to results publications. These measures could improve the transparency, interpretability, and clinical usefulness of the CML trial evidence base.

This study has several limitations. First, trial identification and characterization primarily relied on records from five clinical trial registries. Therefore, unregistered studies, records not captured by the selected registries, and trials with incomplete or outdated registry information may not have been fully represented. Registry entries may also change over time, and some trial characteristics, including phase, target profile, completion status, and study location, were missing or inconsistently reported. Second, although we supplemented registry searches with searches of bibliographic databases using trial registration identifiers, some eligible results publications may not have been identified because of incomplete indexing, absent or incorrect trial identifiers, inaccessible full texts, or insufficient information to confirm linkage with a registry record. Conversely, the identification of a registry-posted result or a publication does not necessarily indicate complete reporting of all prespecified outcomes. The observed rates should therefore be interpreted as estimates of publicly available results rather than definitive measures of publication bias or reporting compliance. In addition, sponsor type was derived from the registry-recorded lead sponsor and was used as a proxy for funding source; thus, it may not fully capture the actual funding arrangements or the role of collaborators. Registered enrollment was also unavailable for one trial. Third, this was a cross-sectional landscape analysis and cannot establish causal associations between trial characteristics and publication. The phase- and drug-specific comparisons were exploratory, and several subgroups had small sample sizes. In addition, individual trials could involve more than one drug; thus, drug-specific comparisons were not mutually exclusive. Although multiple-comparison adjustment was applied, these findings should be interpreted cautiously. Fourth, an important limitation is that target-based analyses were descriptive and based on canonical drug pharmacology rather than mechanisms demonstrated within individual trials. Many TKIs inhibit multiple kinases with overlapping target spectra, and their biologically relevant target engagement may vary according to dose, exposure, treatment combination, disease phase, and patient-specific disease biology. Therefore, comparisons across target categories should not be interpreted as mechanistic evidence or as attributing efficacy, safety, or trial activity to a single kinase target. Future studies integrating kinase-selectivity data, pharmacokinetic exposure, and trial-level biomarker information may provide a more informative assessment of target-specific effects. Finally, the registry records and identified publications did not permit a robust assessment of trial methodological quality, risk of bias, protocol amendments, or adherence to reporting standards, including concordance between prespecified and reported outcomes. Nor could we reliably evaluate comparative treatment efficacy, safety outcomes, or patient-level characteristics. Moreover, registry data alone cannot determine the scientific quality, clinical relevance, or real-world impact of individual trials. Although we conducted an exploratory population-normalized analysis, we could not fully account for cross-country differences in CML incidence, healthcare resources, research expenditure, regulatory environments, drug availability, approval timelines, or trial-registration practices. Therefore, the observed geographical distribution and population-normalized trial activity should not be interpreted as direct measures of national research productivity or clinical need.

## 5 Conclusion

This registry-based analysis included 125 CML-related TKI clinical trials and characterized their geographic distribution, drug and target profiles, clinical development phases, trial status, and publication patterns. Registered trial activity was concentrated in selected countries, BCR-ABL1-targeted agents, and Phase II trials. Among trials completed at least 2 years before the search date, the overall publication rate was 48.1%, indicating incomplete public availability of trial results. These findings support the need for improved registry data completeness, timely publication, and more transparent monitoring of CML-related TKI clinical research.

## Data Availability

The original contributions presented in the study are included in the article/Supplementary Material, further inquiries can be directed to the corresponding authors.
